# The Homœopathic Physician’s Visiting List

**Published:** 1882-11

**Authors:** 


The Homœopathic Physician's Visiting List. By Robert Faulkner, M. D. Price, $2.00. Second edition. Bœricke & Tafel. 1883.

This visiting list is well known. Its distinctive feature is a repertory of some ninety pages. This repertory contains some good hints; but the advice about cutting short typhoid fever is pernicious.

